# Biomimicry at the Landscape Scale: Agent-Based Model Simulating Beaver-Inspired Construction

**DOI:** 10.3390/biomimetics11070515

**Published:** 2026-07-22

**Authors:** Federico Oliva, Jordan Kennedy, Justin Werfel, Karen Lee Bar-Sinai, Amir Degani

**Affiliations:** 1 Civil and Environmental Engineering, Technion—Israel Institute of Technology, Haifa 3200003, Israel; adegani@technion.ac.il; 2 Woods Hole Oceanographic Institution, Woods Hole, MA 02543, USA; jordan.kennedy@whoi.edu; 3 John A. Paulson School of Engineering and Applied Sciences, Harvard University, Cambridge, MA 02138, USA; jkwerfel@seas.harvard.edu; 4 Graduate School of Design, Harvard University, Cambridge, MA 02138, USA; kl_barsinai@gsd.harvard.edu; 5 Technion Autonomous Systems Program, Technion—Israel Institute of Technology, Haifa 3200003, Israel

**Keywords:** complex adaptive systems, landscape architecture, multi-agent systems, ecological engineering, geomorphology

## Abstract

Natural landscape morphology emerges from continuous, reciprocal interactions between biological agents and their physical environment. Despite its broad application across diverse scientific fields, agent-based modeling remains underexplored in the context of non-human geomorphological change. This paper presents a bio-inspired multi-agent framework to investigate how individual animal behaviors, specifically those of the North American beaver, shape adaptive landscapes. To capture dynamic task specialization, we introduce an architecture that abstracts alternating behavioral preferences into two operational states: *Explorers* (focused on resource identification) and *Builders* (focused on localized engineering). Deployed in a dynamic environment characterized by seasonal vegetation mean-reversion and a dynamic hydrological proxy, our targeted parameter sweeps and Monte Carlo replications demonstrate that decentralized stigmergic heuristics drive emergent spatial patterns. Quantitative metric analysis across varying colony sizes shows that while smaller swarms maintain a stable ecological equilibrium, larger populations trigger an apparent non-linear expansion of the hydrological network via active bank erosion. By establishing this foundational framework, this work provides an open-source tool to further explore non-human agency and regenerative strategies in landscape architecture and environmental design.

## 1. Introduction

Landscape morphology is the emergent result of not only geological processes but also continuous, reciprocal interactions between biological agents and their environment. Thus, landscapes are adaptive systems, shaped by environmental change, as well as by the site’s inhabitants, human and non-human alike [1,2,3]. This work extends biomimicry at the landscape scale, emulating both spatial and temporal aspects of design through multi-agent simulations. While Agent-Based Modeling (ABM) has been successfully utilized to simulate human-environment dynamics in urban settings [4,5,6], its application to understanding non-human geomorphological change remains an underexplored frontier.

In this context, ABM provides a structured computational framework for understanding how specific animal behaviors, modeled as Multi-Agent Systems (MAS) [7], influence the development of adaptive landscapes [8,9].

Designing for adaptive landscapes requires tools and methods that are process- and not merely form-based. Because ABM explicitly translates localized, individual behavioral rules into macroscale geomorphological patterns, it provides a suitable framework for simulating, testing, and leveraging these emergent ecological processes. Rather than relying solely on static spatial layouts, an emergent design approach allows for direct interface with dynamic natural forces.

As a clear example, the logic of emergence characterizes the ways North American beavers (*Castor canadensis*) change landscapes. Widely recognized as prolific “ecosystem engineers” [10,11,12], beavers construct an adaptive infrastructure that emerges through iteration and persistence. While traditional research often focuses on the single-dam framework, our approach reframes beaver building behavior within the broader context of the *beaver damming complex* (BDC), a landscape-scale (≈1 $\;{\mathrm{km}}^{2}$) network comprising dams, lodges, trails, canals, scent mounds, and food caches [11,13]. While dams and the resulting ponds are the most visible markers of this engineering, the trail and canal systems that constitute the BDC’s logistical infrastructure remain comparatively understudied [14,15].

To address the lack of dynamic computational tools for non-human geomorphological change, this paper investigates how individual beaver behaviors, coupled with environmental feedback [16], drive the emergence of trail networks and ponds [17,18]. Specifically, the primary contributions of this paper are twofold:

1. We present a novel, open-source simulation suite [19] that integrates real-world Geographic Information Systems (GIS) data (specifically Digital Elevation Models (DEMs) and aerial RGB imagery [20,21]) with an active MAS architecture.
2. We introduce biologically plausible behavioral policies that successfully give rise to large-scale topological features qualitatively corresponding to the search trails and persistent ponds observed in natural systems.

The remainder of this paper is organized as follows: Section 2 presents the Materials and Methods, detailing the environmental initialization pipelines, the dynamic simulation, the MAS architecture, and the biomimetic behavioral policies. Section 3 presents the Results and Discussion, illustrating how low-level agent policies drive emergent landscape morphologies, and evaluating the computational and ecological implications of the framework. Finally, Section 4 provides the Conclusions, explicitly outlining the study’s limitations and our roadmap for future empirical validation. Supplemental mathematical derivations and empirical data calibration steps are detailed in the Appendix A and Appendix B.

## 2. Materials and Methods

As introduced in Section 1, this work develops a multi-agent simulation to investigate the behavioral mechanisms underlying trail formation and canalization in beaver-like agents [22,23]. The simulation is built using the Mesa framework [24] and can be deployed separately on both DEMs and RGB aerial imagery of real landscapes. The test cases presented in this work utilize data obtained from the National Oceanic and Atmospheric Administration (NOAA) [25] and the United States Geological Survey (USGS) [26]. These datasets are supplemented by experimental data acquisition campaigns conducted across three US-based sites: Acadia National Park (Maine) [27], Rumney Marsh Reservation (Massachusetts) [25], and Rumney Ranch (Montana) [28]. Similar to [9], the framework is structured into two primary modules: (1) the *Environment* and (2) the *Agent* [19].

### 2.1. Dynamic Environment Backend via Empirical Geospatial Data

To ensure the simulation framework is applicable to various real-world scenarios, it is designed to be data-agnostic. The BeaversEnvironmentBackend class transforms raw geographic data into the standard N×M matrix required for the simulation. Instead of a single integrated map, the framework provides two independent initialization pipelines (DEM or RGB), chosen based on the required structural features and data availability:

- **DEM Pathway:** Utilizes Digital Elevation Models to derive the environmental matrix. This pathway is ideal for capturing macroscopic topographic gradients and establishing hydraulic constraints (as used in our test cases at 0.5–1.0 m resolution). Because it relies on elevation data, the resulting spatial field exhibits smooth gradients.
- **RGB Pathway:** Utilizes high-resolution optical imagery (e.g., aerial captures at 1.4–1.7 cm/pixel) to extract biomass distribution via spectral indices (e.g., Excess Green). This pathway is sensitive to high-frequency spatial granularity (e.g., micro-canopy density or existing foraging trails) that elevation models inherently miss.

Supporting independent DEM and RGB pipelines allows agent deployment regardless of available field data. To maintain the methodology’s focus on multi-agent behavior, vision protocols, thresholds, and data calibration steps are detailed in Appendix A.

### 2.2. Environmental Representation and Dynamics

The environment is represented as a 2D matrix $E\in R^{N\times M}$, where each cell (x,y) is associated with a scalar state $v_{x,y}$ representing the local vegetation quality. The backend linearly rescales the vegetation values obtained from the initial DEM/RGB pre-processing to a user-defined interval $\left[V_{\min},V_{\max}\right]$, with a default of [−10,10]. The resulting state’s values are interpreted as follows:

- $v_{x,y}<0$: Water-dominant cells, where lower values correlate with increased depth;
- $v_{x,y}\in \left[0,3\right)$: Bare terrain or soil, representing low-resource regions;
- $v_{x,y}\in \left[3,7\right)$: Grasslands or low-density vegetation;
- $v_{x,y}\in \left[7,10\right]$: High-density vegetation (e.g., bushes or trees).

E acts as the primary sensory input for the agents’ navigation policies and, conversely, is modified by their constructive and destructive behaviors.

#### 2.2.1. Vegetation Dynamics and Seasonal Mean-Reversion

Vegetation dynamics are modeled as a mean-reverting stochastic process reflecting seasonal growth, foraging depletion, and environmental noise. This cycle is driven by a right-skewed normal distribution capturing rapid vernal acceleration, a summer plateau, and gradual autumnal decay. The formal derivation of the governing Stochastic Differential Equations (SDEs) and their discrete-time bounds are detailed in Appendix B.

#### 2.2.2. Baseline Vegetation Dynamics Validation

**Figure A2:**
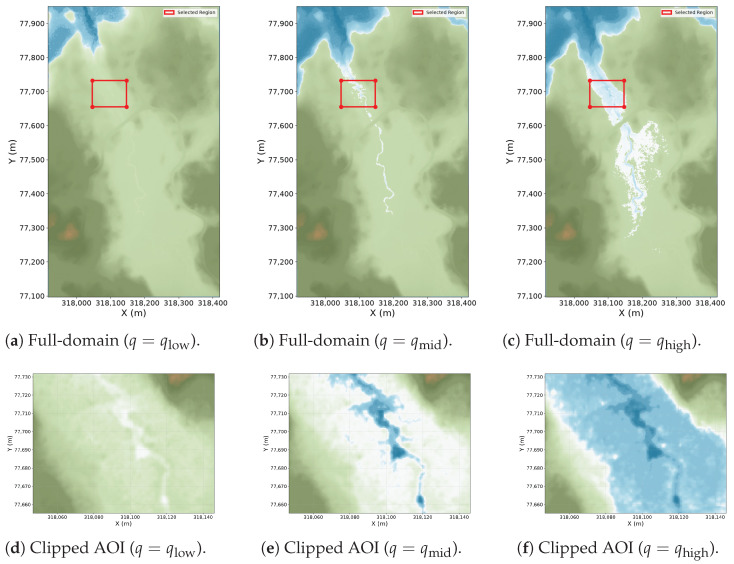
Comparison of percentile threshold sensitivity across the Acadia DEM (0.5 m resolution). The top row (**a**–**c**) displays the full-domain water extraction for low, middle, and high percentiles. The bottom row (**d**–**f**) shows the corresponding AOIs. The colorbar follows the convention of Figure A1.

**Figure A3:**
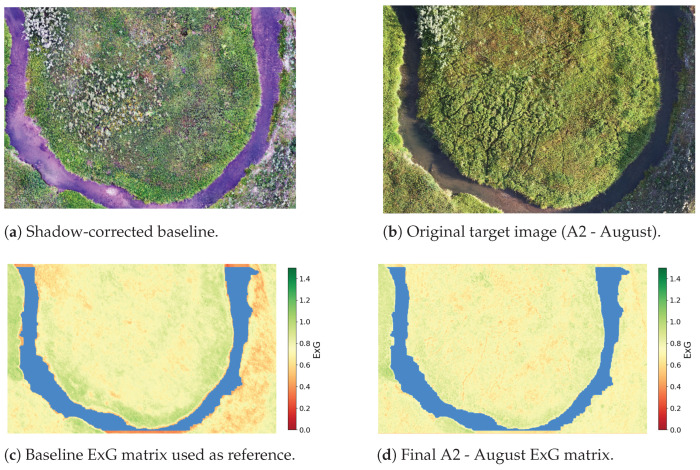
Rumney Ranch A2 RGB preprocessing. (**a**) Aggregated baseline for noise and shadow attenuation; (**b**) target acquisition displaying illumination gradients; (**c**) baseline vegetation-quality reference; (**d**) final normalized matrix highlighting high-frequency trail features in the bottom-left.

Environmental dynamics were validated on the Acadia and the Rumney Ranch datasets. In the Acadia case study, the environment was initialized using the DEM-derived matrix from Figure A2e, whereas in the Rumney Ranch case study, Figure A3c was used. Both images were scaled to [−10,10]. The vegetation parameters were calibrated to reflect a seasonal cycle characterized by high local stochasticity and a stable global carrying capacity. Parameter mappings are detailed in Table A1. For validation, the baseline grass growth rate is set to $\gamma =5\times 10^{-4}\;h^{-1}$ within interval [0,8], with a stochastic scaling factor $\sigma_{0}=0.5$ for local variability and a mean-reversion gain $k=5\times 10^{-4}$. The seasonal skew-normal distribution uses a peak day ξ=90, window ω=50, and skew α=5.

**Figure 1:**
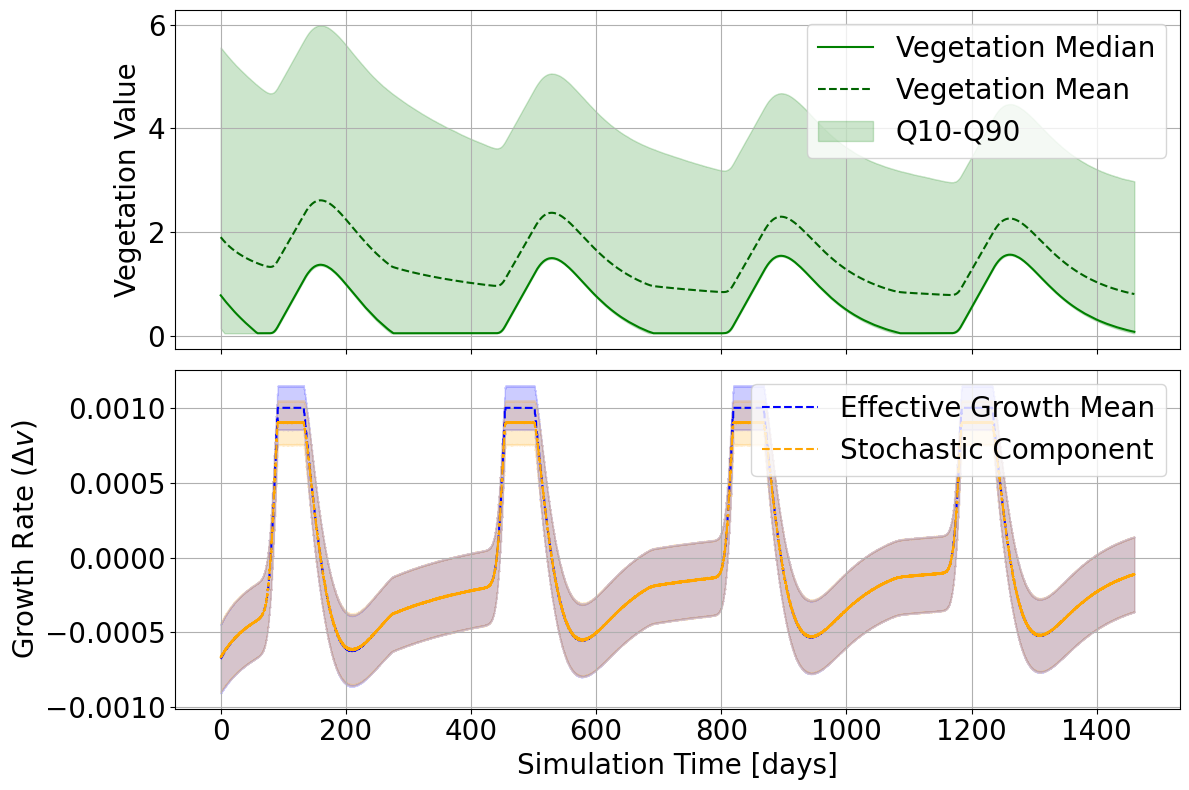
Validation of passive vegetation dynamics on the unforced Acadia site. (**Top panel**): Global vegetation mean, median, and shaded 10th–90th percentile band capturing high spatial variance. The elevated mean relative to the near-zero median indicates a heterogeneous landscape of expansive low-density terrain interspersed with dense peaks. (**Bottom panel**): Bounded, right-skewed growth rate (blue) and local stochastic component (orange), where the seasonal phase shift reflects the underlying biomass integration process.

**Figure 2:**
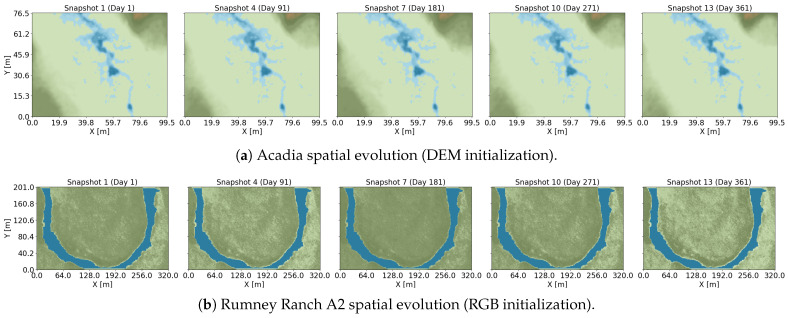
Unforced spatial evolution of the environmental matrix E over one-year simulation. Shades of green-brown describe gevetation, water in blue (**a**) The DEM-based Acadia site. (**b**) The RGB-based Rumney Ranch A2 site.

Let’s consider the Acadia case study: an unforced 1440-day baseline simulation verifies that the environment maintains stable, bounded annual oscillations (Figure 1). Driven by the seasonal PDF, the growth rate (bottom panel) plateaus near day 100, while the integrated vegetation biomass (top panel) peaks near day 160. Mean-reversion prevents state divergence despite high local stochasticity. The positive skewness, with the mean tracking above the median, reflects a heterogeneous landscape of localized, dense patches within expansive barren terrain. Figure 2 compares the Acadia and Rumney Ranch case studies at 30-day intervals, showing how spatial patterns vary by pipeline: the Acadia DEM site (Figure 2a) displays smooth macroscopic gradients, whereas the Rumney Ranch RGB site (Figure 2b) exhibits high-frequency granularity and texture.

Crucially, this evaluation does not benchmark the ecological validity of topographic versus optical inputs, which serve different purposes. Instead, it confirms that the underlying stochastic mean-reversion engine remains stable across initialization pipelines, establishing a data-agnostic architecture. This structural divergence directly informs our subsequent experimental design. We leverage smooth DEM environments (e.g., Rumney Marsh) to evaluate macro-scale routing where topographic gradients and slope are the primary driving constraints. Conversely, we utilize high-resolution RGB environments (e.g., Rumney Ranch) to track micro-scale emergence, such as localized foraging trails and pond expansion, where canopy granularity is strictly necessary.

#### 2.2.3. Hydrological Dynamics and Channel Deepening

Complementing the regenerative vegetation cycle, the aquatic regions of the environment undergo a deterministic, passive-dynamic process that simulates continuous hydrological erosion. Within these defined channels, the state of water-cells is updated at each simulation step according to:

(1) $$v_{x,y}\left(t+\Delta t\right)=\max\left(v_{x,y}\left(t\right)-\nu_{r}\cdot I_{\left[c,d\right]}\left(v_{x,y}\left(t\right)\right),\;c\right),$$

where $\nu_{r}$ represents a constant channel deepening rate, and the indicator function $I_{\left[c,d\right]}$ restricts this erosive process exclusively to the active hydrological interval [c,d]. To prevent unbounded deformation, the landscape state is clipped at depth boundary *c*; variables and state-space coefficients are summarized in Table 1. This water representation is a *conceptual geomorphological proxy* for agent interaction rather than a hydrological simulation. The model omits flow accumulation, hydraulic connectivity, mass conservation, or dam-induced backwater effects handled by solvers like ArcGIS [29] valley-line delineation. Instead, it provides a spatial boundary where localized, agent-induced erosion continuously degrades riverbanks. This drives conceptual water ingress that progressively fractures adjacent terrain into auxiliary stream networks and ponds, mimicking BDC expansion.

#### 2.2.4. Stigmergic Ledger and Topological Constraints

To support decentralized multi-agent coordination, the environment module maintains a secondary spatial ledger, parallel to the primary state matrix E. This *visitation matrix*, denoted as $V\in N^{N\times M}$, tracks a decaying history of all agents across the grid. V serves as the basis for the stigmergic trailing, enabling agents to reinforce foraging corridors without requiring direct communication. Furthermore, the agent-induced canalization is governed by environmental constraints. As we will better describe in Section 2.3, when an agent harvests resources from a cell, it subtracts a discrete quantity of vegetation Δv from the local state $v_{x,y}$. However, to avoid the spontaneous formation of isolated puddles, the environment dictates that an agent can only transition a terrestrial cell into a water cell ($v_{x,y}<0$) if the target cell lies within the Moore neighborhood (D8) of an existing water body. This localized constraint serves as a physical law of the simulation grid, ensuring that agents’ destructive actions lead to the continuous expansion of existing river networks.

### 2.3. Biomimetic Agent Architecture and Control Policies

The multi-agent system is modeled as a decentralized swarm of hybrid dynamical entities operating within the environmental matrices. To bridge the gap between biological observation and computational modeling, the agent architecture is grounded in two fundamental abstractions regarding energetic costs and spatial awareness.

**Assumption** **1** (Backpack and Energetic Proxy)**.** *Beavers transport collected materials using their mouths and forepaws, effectively limiting the volume they can move in a single trip. In the simulation, this is abstracted as a scalar, $L_{\max}$, representing the carrying capacity. This parameter also serves as a proxy for the energetic cost of foraging; a higher $L_{\max}$ indicates a more energetic agent capable of longer excursions before returning to a storage site.*

**Assumption** **2** (Localized Mapping)**.** *Although natural beavers require time to learn new environments, the simulations herein operate on multi-year time scales. Thus, all behavioral and harvesting decisions are strictly constrained to a localized sensory horizon (e.g., a 5-m radius).*

#### 2.3.1. Agent State Representation and Dynamics

Building upon these principles, the comprehensive internal state of the *i*-th agent (robot_beavers_backend.py) at discrete time step *t* is defined by the tuple:

(2) $$S_{i}\left(t\right)=\langle p_{i}\left(t\right),\;v_{i}\left(t\right),\;L_{i}\left(t\right),\;M_{i}^{env}\left(t\right),\;M_{i}^{vis}\left(t\right)\rangle$$

where $p_{i}\left(t\right)\in R^{2}$ and $v_{i}\left(t\right)\in R^{2}$ represent the continuous 2D position and velocity vectors. The scalar $L_{i}\left(t\right)\in \left[0,L_{\max}\left(t\right)\right]$ tracks the current harvested biomass load. The matrices $M_{i}^{env}\left(t\right)\in R^{N_{env}\times N_{env}}\subseteq E$ and $M_{i}^{vis}\left(t\right)\in R^{N_{vis}\times N_{vis}}\subseteq V$ represent the agent’s localized perceptions of the global environment E and the stigmergic visitation ledger V, respectively. On both x- and y-axes, the movement of the agent is governed by a decoupled double-integrator model, incorporating inertial mass *m* and viscous friction μ. For a single spatial dimension, letting $z_{t}={\left[p_{t},v_{t}\right]}^{⊤}$, the discrete-time state-space evolution is formulated as:

(3) $$z_{t+\Delta t}=Az_{t}+Bu_{t},$$

where $u_{t}$ is the control force derived from the navigation policy, and the matrices are:

(4) $$A=\left[\begin{array}{cc} 1 & \Delta t\\ 0 & 1-\frac{\mu \Delta t}{m} \end{array}\right],\;B=\left[\begin{array}{c} 0\\ \frac{\Delta t}{m} \end{array}\right].$$

While the inertial mass dampens the transient acceleration phase during movement, the linear dynamics are augmented with a non-linear hybrid stopping condition: if the control input is zero ($u_{t}=0$), the velocity state is instantaneously truncated to zero ($v_{t+\Delta t}=0$). This hybrid formulation eliminates inertial drift, allowing the agent to execute precise stigmergic actions (such as excavation) without overshooting the intended coordinate.

#### 2.3.2. Hierarchical Cognitive Architecture: Tasks and Actions

**Figure 3:**
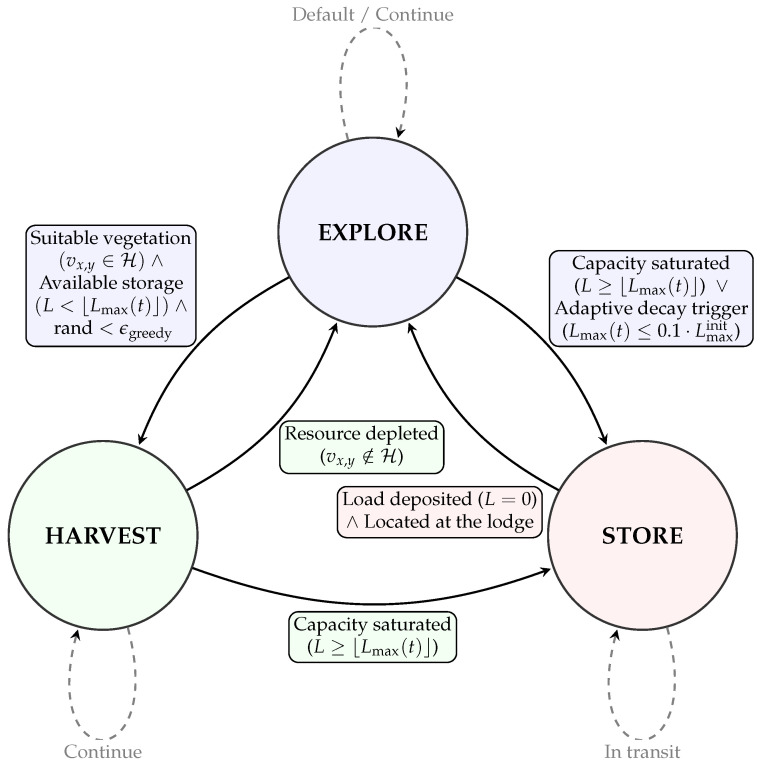
State transition diagram of the agent’s atomic Finite State Machine. The policy is evaluated sequentially at every temporal step Δt. Transitions are governed by internal capacity (*L*), local environmental quality ($v_{x,y}$), stochastic exploration ($\epsilon_{\mathrm{greedy}}$), and adaptive parameter decay.

The agent’s decision-making is structured as a two-layered hierarchical state machine. This decouples the overarching biological objectives (the Strategic Layer, see Figure 3) from their discrete physical execution within the simulated environment (the Tactical Layer).

##### Strategic Layer: The Task FSM

The overarching behavioral intent is governed by an atomic FSM evaluated at every temporal increment Δt. Because the agent relies on the localized spatial patch $M_{i}^{env}\left(t\right)$ and on the stigmergic visitation ledger $M_{i}^{vis}\left(t\right)$, it operates reactively without long-horizon path planning.

The deterministic state transitions are evaluated sequentially:

- **Store:** Triggered if the agent’s capacity is saturated ($L_{i}\left(t\right)\geq \left\lfloor L_{\max}\left(t\right)\right\rfloor$), or if adaptive parameter decay forces the maximum capacity below 10% of its initial value $L_{\max}^{\mathrm{init}}$, compelling a return to base.
- **Harvest:** Triggered if capacity is available, local resources are valid ($v_{x,y}\in H\subseteq \left[V_{\min},V_{\max}\right]$), and the agent is not on a lodge site. To introduce natural behavioral variance, this state is gated by a stochastic ϵ-greedy condition ($\mathrm{rand}<\epsilon_{\mathrm{greedy}}$).
- **Explore:** The default spatial routing state is initialized when immediate resources are depleted, invalid, or stochastically bypassed.

##### Tactical Layer: The Action FSM

Once a strategic task is assigned, the Tactical Layer dictates how it is computed. The overarching tasks are decomposed into three actions: move, remove_vegetation, or idle. Specifically, an EXPLORE task triggers a move action, whereas a HARVEST task triggers a remove_vegetation action upon reaching the target coordinate. While the environmental framework is architecturally capable of supporting natural circadian rhythms (e.g., forcing a global idle state during diurnal/nocturnal resting phases), the current agent implementation intentionally abstracts this cycle to isolate morphological emergence. Because resting phases effectively act as a linear temporal scaling factor, their inclusion would introduce redundant computational complexity without fundamentally altering the emergent spatial topology. Consequently, when comparing the simulated multi-agent movement rates with biological tracking data, the continuous 24 h simulation time must be interpreted strictly as aggregated “active foraging hours” rather than absolute chronological time.

#### 2.3.3. Strategic Target Selection and Agent Roles

Once the strategic layer triggers an EXPLORE or HARVEST task, the agent selects a waypoint $p^{*}$ within its sensory patch $M_{i}^{env}\left(t\right)$. While literature explicitly classifying rigid beaver castes is sparse, empirical wildlife observations indicate that individuals exhibit clear behavioral preferences, naturally allocating unequal time to foraging, building, territory defense, or parenting. Biologically, these represent flexible behavioral states rather than the fixed, genetic castes of eusocial insects. To abstract these preferences efficiently without overcomplicating the decision matrix, the framework introduces two profiles that alter how agents evaluate local spatial utility U(x,y): *Explorers*, prioritizing foraging (seeking dense vegetation), and *Builders*, prioritizing engineering (seeking bare terrain or water boundaries for construction and transport).

Such a distinction affects the agents’ exploration policy. In fact, the framework provides a profile-based user-configurable parameter (exploration_map) to toggle stigmergic coupling. Let’s recall the global stigmergic ledger $V\in R^{N\times M}$. To ensure that the magnitude of the stigmergic record remains comparable to the vegetation state during utility calculations, the environment continuously normalizes the entire ledger into a bounded matrix $\tau \in [0,V_{\max}]$, as specified in Equation (10) below.

- **Mode 1: Resource-Driven (****vegetation_quality****):** Navigation relies exclusively on the primary environmental state. *Explorers* seek pristine foraging grounds by scaling utility directly with resource density: $U_{\exp}\propto {\left(\varepsilon +v_{x,y}\right)}^{2}$. Conversely, *Builders* seek construction sites (e.g., bare terrain or water boundaries) by mathematically inverting the resource gradient: $U_{\mathrm{bld}}\propto {\left(\varepsilon +v_{x,y}\right)}^{-2}$.
- **Mode 2: Stigmergically Coupled (****vegetation_visits****):** Navigation integrates the secondary spatial ledger to reinforce established corridors. For *Explorers*, the resource utility is amplified by historical traffic: $U_{\exp}\propto \left(\varepsilon +\tau_{x,y}\right)\left(\varepsilon +v_{x,y}\right)$. For *Builders*, the inverted gradient is multiplied by the visitation density: $U_{\mathrm{bld}}\propto \left(\varepsilon +\tau_{x,y}\right)/\left(\varepsilon +v_{x,y}\right)$.

**Remark** **1** (Stigmergic Coordination in Target Selection)**.** *The stigmergically coupled evaluation mode marks the first active integration of stigmergy into the agent’s decision-making pipeline. In this mode, builders do not merely seek arbitrary bare terrain; the visitation term $\left(\varepsilon +\tau_{x,y}\right)$ actively guides their exploratory efforts along highly traversed corridors.*

#### 2.3.4. Tactical Execution: Dynamic Control and Obstacle Avoidance

After computing the spatial utility for all valid cells in the immediate neighborhood, the agent calculates the local utility gradient $\Delta U_{j}$. The probability of selecting a specific neighboring coordinate *i* as the next waypoint $p^{*}$ is governed by a Softmax distribution:

(5) $$P\left(i\right)=\frac{\exp(\Delta U_{i})}{\sum_{j}\exp\left(\Delta U_{j}\right)}.$$

When the tactical layer authorizes a move action, the agent must physically navigate to the sampled waypoint $p^{*}$. The control effort $u_{t}$ driving the double-integrator dynamics (Equation (3)) is generated on each axis by a closed-loop Artificial Potential Field (APF) controller, augmented with a Proportional–Integral–Derivative (PID) goal-seeking law:

(6) $$u_{t}=\underset{\mathrm{Attractive}\;\mathrm{Force}}{\underset{︸}{\left(K_{p}e_{t}+K_{i}\int e_{t}\;dt+K_{d}\frac{de_{t}}{dt}\right)}}-\underset{\mathrm{Repulsive}\;\mathrm{Force}}{\underset{︸}{\beta \nabla M_{\mathrm{repulsive}}\left(p_{t}\right)}}+\underset{\mathrm{Water}\;\mathrm{Drift}}{\underset{︸}{\lambda \hat{f}\left(p_{t}\right)}},$$

where $e_{t}=p^{*}-p_{t}$ is the positional error vector, β is a scaling weight applied to the environmental penalty gradient, and λ represents the strength of stream currents.

To accurately reflect terrestrial traversal costs, the repulsive field $M_{\mathrm{repulsive}}$ is dynamically constructed based on the agent’s immediate local neighborhood. Similar to the exploration utility, this penalty supports two configurable modes:

- **Resource-Driven Friction (****vegetation_quality****):** Evaluated as $M_{\mathrm{repulsive}}\propto v_{x,y}$. Dense, high-cost patches naturally generate strong localized repulsive gradients, forcing the trajectory to dynamically weave around barriers.
- **Stigmergically Mitigated Friction (****vegetation_visits****):** Evaluated as $M_{\mathrm{repulsive}}\propto v_{x,y}/\left(1+\tau_{x,y}\right)$. Historical traversal acts as a physical modifier. Highly visited cells exhibit lowered resistance, simulating trampled vegetation and foraging trails.

*Methodological Note:* To maintain clarity and isolate emergent behaviors, researchers are advised to apply stigmergic coupling exclusively to either the high-level exploration heuristic (Section 2.3.3) or this low-level APF controller, avoiding redundant feedback.

#### 2.3.5. Hydrological Drift and Riparian Biasing

Distinct from the cognitive obstacle avoidance logic governed by $M_{\mathrm{repulsive}}$, the simulation subjects the agent to direct physical hydrological forces. This is represented by the third term in Equation (6), where $\hat{f}$ is an externally defined river flow vector acting upon the aquatic cells of the environment ($v_{x,y}<0$). Because this flow acts as an independent environmental drift, it is not bound by the repulsive weighting factor (β). Consequently, when the agent navigates aquatic cells, the flow vector is superimposed directly onto the final control effort $u_{t}$. This current assists downstream and resists upstream movement.

#### 2.3.6. Distance-Gated Local Minima Resolution

A well-documented flaw of standard APF controllers is the tendency for agents to stall in local spatial minima, particularly when a target is adjacent to a strong repulsive obstacle. To resolve this without incurring the computational overhead of global path-planning algorithms (e.g., A* or RRT), the framework uses a distance-gated terminal attraction phase. The repulsive field is strictly evaluated only when the spatial error norm exceeds a critical threshold (e.g., ${∥e_{t}∥}_{2}>4.0$ pixels). Once the agent breaches this proximity radius, $\nabla M_{\mathrm{repulsive}}$ is instantaneously forced to zero. The controller drops the obstacle-avoidance constraint and relies solely on the purely attractive PID law to converge to the final target. This effectively allows the agent to “push through” boundary friction to reach its localized objective, mimicking a biological agent as it finalizes its approach to a specific resource.

#### 2.3.7. Environmental Modification

When a HARVEST task gets to its spatial target, the tactical layer transitions the agent from locomotion to excavation by executing the remove_vegetation action. This process facilitates a direct state transfer between the environmental matrix E and the agent’s internal resource capacity. At the target coordinate (x,y), the agent extracts a discrete quantum of biomass Δv, synchronously updating both the environmental state and its internal load:

(7a) $$v_{x,y}\left(t+\Delta t\right)=v_{x,y}\left(t\right)-\Delta v\;,$$

(7b) $$L_{i}\left(t+\Delta t\right)=L_{i}\left(t\right)+\Delta v\;.$$

As introduced in Section 2.2.4, the environment module enforces a strict topological constraint: an agent can only transition a terrestrial cell into an aquatic cell ($v_{x,y}<0$) if the target coordinate lies within the immediate Moore neighborhood (D8) of an existing water body. The ecological rationale is rooted in fluid continuity; water ingress relies on the erosion of existing channel banks rather than spontaneous upwelling. This forces the emergent network to expand contiguously, directly mirroring natural BDC topology.

#### 2.3.8. Writing to the Stigmergic Ledger

The stigmergic trail mechanism is governed by a three-step update cycle at each time step *t*: (1) agent deposition, (2) environmental decay, and (3) global normalization.

1. In parallel with physical excavation, each agent navigating the grid deposits a discrete traffic marker Δτ onto the ledger at its current coordinate (x,y):
  (8) $$V_{x,y}\leftarrow V_{x,y}+\Delta \tau$$
2. The ledger values decay to simulate the fading of trails:
  (9) V←η·V,withη∈(0,1)
3. Finally, the entire ledger is normalized to the vegetation scale to yield the local visitation record $\tau_{x,y}\left(t+\Delta t\right)$:
  (10) $$\tau_{x,y}\left(t+\Delta t\right)=\frac{V_{x,y}-\min\left(V\right)}{\max(V)-\min(V)}\cdot V_{\max}$$
  Division by zero in the normalization step is avoided programmatically because the simulation suite enforces strict bounds during ledger initialization, ensuring that max(V)>min(V) at all times t≥0. For more details, please refer to Appendix A. Once processed through this cycle, the normalized record $\tau_{x,y}$ reduces localized friction for subsequent agents (as formalized in Section 2.3.4) and actively shapes the colony’s probabilistic exploration gradients (Section 2.3.3), closing the decentralized feedback loop.

#### 2.3.9. Adaptive Foraging and Parameter Decay

Biological systems rarely operate under static conditions; prolonged failure to acquire resources can induce behavioral shifts. To capture this reality, the architecture implements a time-dependent parameter-decay loop driven by the agent’s foraging efficiency. If the frequency of successful harvest events falls below a critical threshold, the agent registers a stagnation state. To resolve this, the framework applies a multiplicative decay to a pair of configurable behavioral parameters: the harvest interval (H) and the maximum carrying capacity ($L_{\max}$). Each decaying parameter induces a specific tactical shift in the agent’s logic and its resulting impact on the simulated landscape:

- **Harvest Interval (H):** As stagnation persists, the acceptable bounds of vegetation density systematically expand. Behaviorally, the agent lowers its foraging standards out of simulated desperation. Environmentally, this accelerates the depletion of marginal canopy zones that the swarm would normally bypass.
- **Carrying Capacity ($L_{\max}$):** A gradual reduction in $L_{\max}$ serves as a biological proxy for metabolic exhaustion. A shrinking capacity lowers the threshold required to trigger the STORE task (Section 2.3.2), terminating excursions and compelling a premature return to the lodge to prevent starvation.

Upon a successful sequence of harvest actions, the agent breaks the stagnation cycle, and all three parameters are instantaneously reset to their initial baseline values. To facilitate reproducibility and establish a parameter space for subsequent experimental validation, the core user-definable variables governing the multi-agent behavioral logic, dynamics, environmental dynamics, and main modeling choices, are reported in Table 2, Table 3, Table 4, and Table 5, respectively.

## 3. Results and Discussion

To validate the framework, the architecture was deployed across two environments to isolate specific behaviors. First, the Rumney Marsh dataset evaluates low-level kinematics and hydrological drift mechanics using its well-defined stream networks. Second, the Rumney Ranch dataset is used to investigate macroscopic swarm dynamics and stigmergic trail formation. This computationally tractable site balances long-term simulation scale with high-resolution RGB imagery, establishing a baseline for future cross-validation against actual biological trails.

### 3.1. Agent Dynamics and Navigation Policies

This subsection evaluates the agent’s movement capabilities on the Rumney Marsh dataset, focusing on integrating the double-integrator physics engine with the distance-gated APF controller. First, we establish how the agent’s velocity scales dynamically with both its cognitive spatial horizon and the chosen integration time. We then analyze the necessary tradeoff between computational efficiency and biomimetic accuracy.

#### 3.1.1. Simulation Setup

To isolate the routing dynamics from the multi-agent coordination, a single-agent environment (n=1) was considered. Active biomass removal (Δv=0) and seasonal vegetation growth were disabled, forcing the agent to navigate a static spatial utility field U(x,y). Similarly, adaptive parameter decay was deactivated to ensure the agent’s cognitive thresholds remained constant throughout the simulation. Furthermore, stigmergic coupling was explicitly disabled (exploration_map = vegetation_quality). This setup isolated the agent’s response to primary environmental gradients and prevented “stigmergic self-attraction,” namely, an artifact in which an isolated agent becomes trapped in self-reinforcing orbital loops by continuously following its immediate traversal history. Furthermore, the temporal decay of the visitation ledger was set to zero to preserve a cumulative spatial memory of the entire trajectory, as trail fading becomes a relevant control dynamic only during multi-agent stigmergic coordination. Finally, the river flow was set to zero to eliminate the influence of passive hydrological transport.

To govern the double-integrator dynamics, the agent’s physical parameters were established to reflect the empirical biomechanics and environmental resistance experienced by *Castor canadensis*. Specifically, the inertial mass was set to m=20.0 kg, coupled with a viscous friction coefficient of μ=3.0. The continuous control effort driving this physical model was regulated by the closed-loop APF controller (Equation (6)). To ensure numerical stability and realistic decision-making, the system’s state-space integration time step was decoupled to dt=0.1 h (equivalent to 6-min cognitive intervals).

The controller’s proportional, derivative, and integral gains were tuned to $K_{p}=200.0$, $K_{d}=300.0$, and $K_{i}=10^{-5}$, respectively. During traversal, terrain negotiation was dictated by a localized repulsive weight (β=0.2), calculated over a two-cell spatial neighborhood and based on the underlying vegetation quality matrix. Finally, to counteract the asymptotic slowdown inherent to standard APF algorithms near target destinations, a terminal positional accuracy threshold of 0.5 m was enforced.

#### 3.1.2. Cognitive Spatial Horizons and Velocity Modulation

This section evaluates the impact of the agent’s cognitive spatial horizon on its macroscopic velocity. To isolate the dynamics’ impact on the agent’s decision-making cycle, the action-selection mechanism was constrained using an ϵ-greedy policy with ϵ=0.5. This configuration enforces a symmetric probability distribution between exploration and harvesting. Because the harvest state acts as a stationary temporal delay in this specific experimental context (Δv=0), the agent is forced into a stop-and-go pattern, spending approximately 50% of its time in an idle configuration. The agent’s ability to explore the surrounding area is governed by the radial depth of its spatial horizon, i.e., $N_{vis}$.

**Figure 4:**
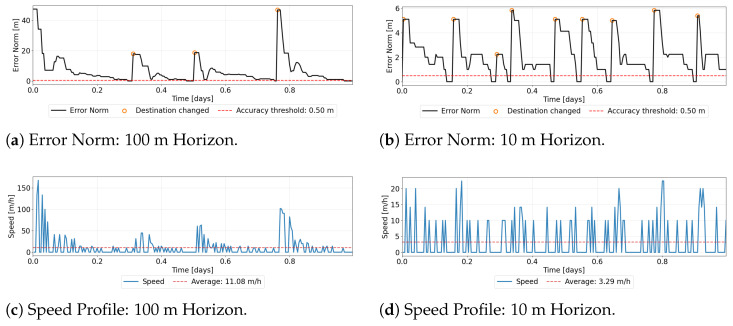
Comparative kinematic analysis of the APF controller across two spatial horizons (24 h Rumney Marsh simulation). Row 1 illustrates the error norm, demonstrating stable asymptotic convergence without discretization instability. Row 2 details the associated speed profiles—the expanded horizon enables peak velocities of 160 m/h (**c**), whereas the constrained 10 m horizon mechanically caps peak speeds at ∼20 m/h (**d**).

To first establish the physics engine’s mechanistic capabilities, a baseline simulation was conducted with a theoretical cognitive spatial horizon of 50 m radially (100 m total diameter). The kinematic impact of this extended horizon is captured in Figure 4a,c. The control error norm (Figure 4a) shows that destination updates frequently project targets up to 50 m away, with the double-integrator converging on the 0.50 m accuracy threshold. Notably, minor transient oscillations are observable during this terminal convergence. This behavior is a deliberate consequence of the controller’s tuning; an aggressive PID configuration was explicitly selected to maximize acceleration, ensuring a rigorous assessment of the system’s absolute high-speed traversal behavior. Granted sufficient spatial runway by this expanded horizon, the physical engine reaches peak transit velocities exceeding 150 m/h (Figure 4c). Due to the ϵ-greedy policy, the agent’s effective macroscopic velocity averages ≈11 m/h.

#### 3.1.3. Kinematic Profiles of Localized Terrestrial Foraging

To transition from theoretical to biomimetic traversal, the spatial horizon was subsequently restricted to 5 m radially (10 m total diameter). This heavily constrained perimeter reflects the localized sensory capabilities of *C. canadensis* during terrestrial motion. The kinematic bottleneck induced by this restricted decision horizon is demonstrated in Figure 4b,d. Because the physical engine is confined to short-distance transits (never exceeding 6 m, Figure 4b), the agent remains within the transient acceleration and deceleration phases of the APF controller. Consequently, the agent never achieves its biomechanical top speed; velocity peaks are capped at approximately 20 to 25 m/h (Figure 4d). When combined with the 50% idling from the ϵ-greedy policy, this kinematic truncation restricts the agent’s macroscopic displacement rate to ≈3 m/h. In nature, free-ranging beavers achieve a higher overall displacement rate (≈536 m/h) by leveraging efficient aquatic routes during a compact ≈ 9.7 h nocturnal window [30].

Because our framework explicitly models a land-only, continuous 24 h operational cycle without rest, spreading the daily foraging footprint across a full, nonstop diurnal loop dilutes the hourly velocity. While this 3 m/h speed is highly conservative compared to true biological benchmarks, it establishes a safe, numerically feasible lower bound for the simulation while still capturing the cautious terrestrial locomotion and localized patch exploitation typical of foraging *C. canadensis*.

### 3.2. Computational Tractability and Temporal Upscaling

While the integration interval dt=0.1 h replicates the micro-kinematics of localized foraging, it introduces a computational bottleneck. A single agent requires 7 s to simulate a 24 h period. When extrapolated to multi-agent swarms operating over annual timescales, this overhead becomes prohibitive. To facilitate large-scale simulations, the framework needs temporal upscaling, increasing the integration step to dt=1.0 h. However, increasing the integration step alters the stability of the discrete-time engine. Maintaining the baseline velocity (v≈11 m/h) at dt=1.0 h results in discrete positional updates of 11 m per tick. This exceeds the agent’s sensory radius ($r_{max}=5$ m), causing it to overshoot local waypoints. To respect spatial motion boundaries, the allowable velocity must satisfy $v_{max}\leq r_{max}/dt$, capping transit speeds at 5 m/h.

**Figure 5:**
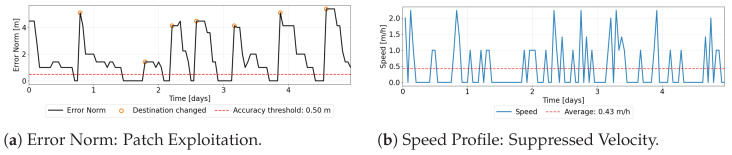
Kinematic profile of the temporally upscaled framework (dt=1.0 h) over a 5-day simulation on the Rumney Marsh site. (**a**) The control error norm illustrates a reduction in destination updates, with spatial projections bounded within the 5-m horizon. Coupled with the ϵ-greedy idling penalty (ϵ=0.5), it takes 120 h to accumulate the same number of waypoint visits generated in 24 h under the baseline (Figure 4b), confirming the shift to extended patch exploitation. (**b**) The velocity profile shows the effect of the dampened physical parameters ($m=5,\mu =1,K_{p}=0.5,K_{d}=2.0$), which cap the peak velocity at ∼2.2 m/h. This dampening satisfies the spatial motion boundary condition ($v_{max}\leq 5$ m/h), preventing the agent from overshooting the cognitive horizon.

Consequently, the physical dynamics and APF control gains were retuned to improve system damping. Mass and friction were adjusted to m=5 and μ=1, paired with lower control gains ($K_{p}=0.5$, $K_{d}=2.0$, $K_{i}=10^{-5}$). As illustrated in Figure 5, this recalibration restricts the peak velocity to around 2.2 m/h, well below the 5 m/h bound. The error norm verifies this stability, showing that spatial projections remain within the 5-m horizon.

To evaluate the macroscopic impact of this dampening, the simulation duration was extended to 5 days, providing a comparative temporal scale against the baseline model. Because the transit time is extended, and the symmetric action-selection policy (ϵ=0.5) forces the agent into a 1 h idle state during ≈50% of its decision time, the behavioral frequency is bottlenecked. As a consequence, the agent requires 5 days to generate the same number of destination updates achieved in a single day under the baseline (dt=0.1 h), transitioning from rapid micro-transit to extended patch exploitation.

### 3.3. Landscape-Scale Navigation and Topographic Routing

Having established the stability of the physical engine (dt=1.0 h), the macroscopic spatial routing was evaluated. It is critical to distinguish the scope of the APF from the high-level tactical layer. The APF repulsive gradient ($\nabla M_{\mathrm{repulsive}}$) is intentionally near-sighted. It is designed to induce localized swaying to bypass immediate topographic resistance. It does not act as an exclusionary wall. If the tactical layer designates a waypoint deep within a dense thicket, the APF will minimize the traversal cost while ultimately permitting entry.

**Figure 6:**
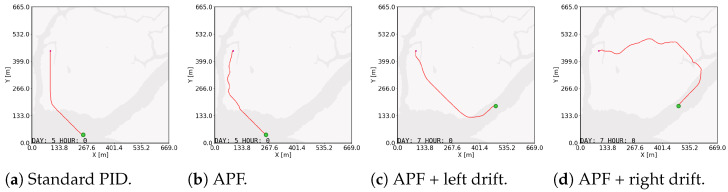
Deterministic spatial routing across a static landscape. Across all panels, the agent’s trajectory is traced in red, originating from the home lodge (green circle) and targeting the terminal destination (magenta circle). (**a**) A pure attractive PID controller forces a biologically unnatural path through high-cost obstacles. (**b**) Activation of the near-sighted APF repulsive field induces localized weaving, allowing the agent to circumnavigate vegetation without abandoning its terminal waypoint. (**c**,**d**) The introduction of an independent aquatic drift vector ($\lambda \hat{f}$) displaces the agent during water crossings, creating pronounced rightward and leftward bows before terrestrial weaving resumes.

To isolate the APF’s pathfinding capabilities, a deterministic control simulation was run. The vegetation removal rate was disabled (Δv=0) to freeze the cost surface, and the agent was assigned a fixed, long-range destination across the environment. Figure 6 illustrates the emergent trajectories under varying control configurations. Under a standard goal-seeking PID controller (Figure 6a), the trajectory follows a biologically unnatural path directly to the destination. Conversely, activating the repulsive field (Figure 6b) fundamentally alters the trajectory. Without preventing the agent from reaching its terminal destination, the repulsive gradients deflect the trajectory, resulting in a localized weaving motion that circumnavigates the densest vegetation areas. Lastly, the framework was subjected to physical hydrological biasing to simulate riparian transit.

Utilizing the independent environmental drift vector ($\lambda \hat{f}$) defined in Equation (6), the agent was forced to cross a water body under two opposing current conditions. When subjected to a leftward drift vector [−1,−1] (Figure 6c), the current displaces the agent off its optimal terrestrial vector, resulting in a pronounced leftward bow during the aquatic crossing.

Once the agent exits the aquatic cells, the drift dissipates, and the trajectory resumes its standard terrestrial pathfinding. Reversing the current to induce a rightward drift [1,1] (Figure 6d) yields the exact mirrored response, pulling the trajectory into a wide rightward arc. This deterministic test confirms that the framework successfully integrates independent physical environmental forces with cognitive terrain avoidance.

### 3.4. Swarm Dynamics: Payload Constraints and Stigmergic Routing

Next, macroscopic swarm dynamics are evaluated on the Rumney Ranch dataset; its RGB imagery serves as a baseline for future comparisons with actual biological trails. Specifically, colony-level spatial organization is evaluated via targeted parameter sweeps across three axes: payload constraint ($L_{\max}$), routing policy (vegetation_quality vs. vegetation_visits), and obstacle avoidance weight (β). To analyze the effects of these parameters on behavioral phenotypes, we track the movement of *Explorers* and *Builders*.

#### 3.4.1. Experimental Setup and Parameter Isolation

To formally isolate the impacts of payload, tactical friction, and routing logic, agents were equally divided into the two phenotypes. A multi-agent colony (n=10) was deployed over a 6-month period. To establish a controlled baseline, seasonal vegetation growth and hydrological drift were suspended. Crucially, both *Explorers* and *Builders* roles were evaluated using an identical harvesting interval (H=[2,6]) and a static vegetation removal rate (Δv=1.0). Furthermore, the visitation decay rate was temporarily suspended (η=1.0), ensuring that the colony’s absolute spatial footprint was permanently captured and eliminating trace-evaporation artifacts. This parameter isolation leaves $L_{\max}$, the utility matrix ($U_{x,y}$), and the low-level repulsive weight (β) as the sole active independent variables. The maximum load was varied between restricted ($L_{\max}=100$) and extended ($L_{\max}=1000$) limits, with no decay rates applied to the payload and harvest interval during sorties. Simultaneously, the continuous APF obstacle avoidance weight (Equation (6)) was swept from a low-responsiveness (β=0.2) to a high-responsiveness (β=0.8) regime to assess how physical terrain friction modulates spatial diffusion. During locomotion, the continuous visitation ledger was updated with Δτ=1. Each scenario utilizes Monte Carlo replication (n=5 random seeds) to improve the statistical robustness of the observed trends.

#### 3.4.2. Quantitative Metrics Formulation

To evaluate the transition from diffuse foraging to structured ecosystem engineering, we extract two macroscopic metrics from the simulation logs: the Spatial Diffusion (D) and the Canalization Ratio (C).

*Spatial Diffusion (D):* Measures cumulative spatial diffusion independent of trail decay (η). For each phenotype r∈{Explorer,Builder}, let $I_{x,y}^{\left(r\right)}=1$ if cell (x,y) has ever been traversed, and 0 otherwise:
  (11) $$D_{r}=\sum_{\forall (x,y)}I_{x,y}^{\left(r\right)}$$
*Canalization Ratio (C):* Quantifies path consolidation by measuring traffic concentrated within primary corridors. On the decaying ledger $\tau_{x,y}^{\left(r\right)}$, active cells are defined as $A_{r}=\left\{\left(x,y\right)|\tau_{x,y}^{\left(r\right)}>10^{-3}\right\}$, yielding a total active traffic volume of:
  (12) $$V_{\mathrm{total}}=\sum_{\left(x,y\right)\in A_{r}}\tau_{x,y}^{\left(r\right)}$$

Let $A_{95}=\left\{\left(x,y\right)\in A_{r}|\tau_{x,y}^{\left(r\right)}\geq \tau_{95}\right\}$ be the highly canalized subset exceeding the 95th percentile trace magnitude $\tau_{95}$. The ratio $C_{r}\in \left[0,1\right]$ measures the traffic fraction concentrated within this top 5% active core:

(13) $$C_{r}=\frac{\sum_{\left(x,y\right)\in A_{95}}\tau_{x,y}^{\left(r\right)}}{V_{\mathrm{total}}}$$

Low $C_{r}$ values reflect diffuse, uncoordinated navigation, whereas high values capture the emergence of persistent, heavily exploited infrastructural highways.

#### 3.4.3. Spatial Diffusion: Payload Tethers and Terrain Friction

**Figure 7:**
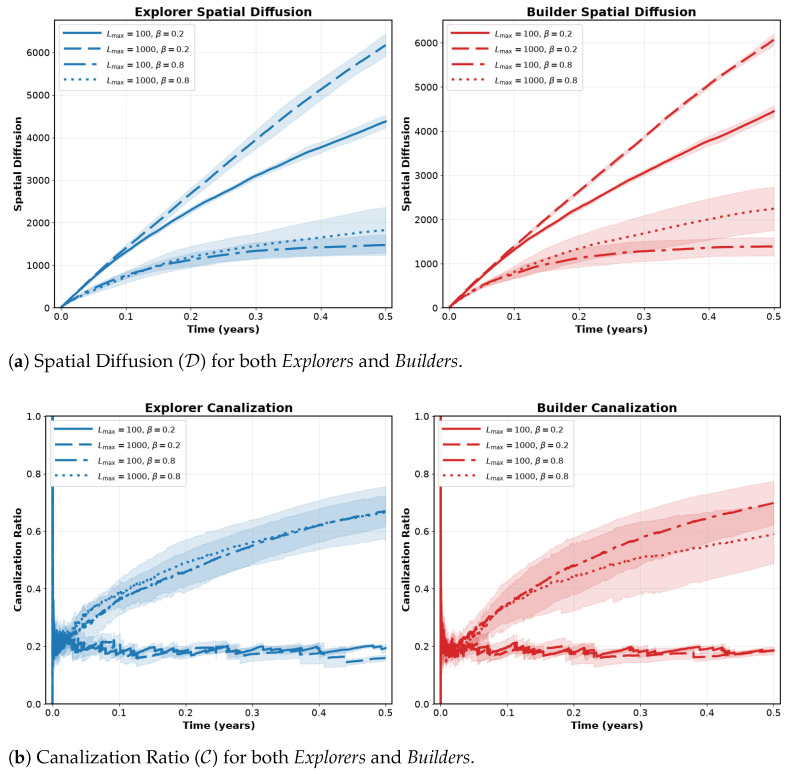
Swarm routing dynamics under resource-driven policy (vegetation_quality). Lines show the Monte Carlo mean (n=5) and shaded bands denote ±1σ variance (shaded areas). (**a**) Spatial Diffusion (D): pconstraints ($L_{\max}=100$) strictly tether agents near the lodge, compressing expansion regardless of terrain friction (β). (**b**) Canalization Ratio (C): high friction (β=0.8) accelerates path consolidation (C→1) via topographic funneling, whereas low friction (β=0.2) permits highly diffuse, non-canalized exploratory topologies.

Under resource-driven routing (vegetation_quality), the Spatial Diffusion (D) is governed by a push-pull dynamic between internal caloric capacity ($L_{\max}$) and external topographic friction (β) (Figure 7a). Crucially, when the payload is strictly constrained ($L_{\max}=100$), high-frequency return trips to the central lodge serve as a physical tether between the two phenotypes. This mechanical restriction prevents the swarm from breaching the immediate perimeter, severely compressing the spatial diffusion regardless of terrain friction. Expanding the payload capacity ($L_{\max}=1000$) drives aggressive footprint scaling, heavily modulated by terrain friction (β). At low environmental sensitivity (β=0.2), agents only mildly take into account local repulsive gradients, carving more direct paths, and compressing inter-trial variance (low σ). Conversely, high sensitivity (β=0.8) introduces rigid topographic barriers that restrict expansion. This high-friction regime inherently amplifies spatial variance (σ) by rendering trajectories highly sensitive to stochastic action selection: minor early deviations compound, deflecting agents down entirely different topographic corridors across Monte Carlo trials.

#### 3.4.4. Vegetation-Induced Canalization

The modulation of terrain friction (β) does not merely restrict spatial diffusion; it actively reshapes the internal network topology. Figure 7b plots the Canalization Ratio (C) of the swarm. Because these specific simulations were restricted to the vegetation_quality mode, agents lack access to the stigmergic memory ledger. Therefore, any observed path consolidation is strictly the result of physical environmental feedback. At β=0.2, the swarm diffuses uniformly, yielding a persistently low, flat Canalization Ratio as agents bypass topographic constraints.

However, at β=0.8, the high sensitivity to terrain friction forces agents to converge on naturally occurring topographic funnels. Crucially, as pioneer agents traverse these paths of least resistance, their obligate harvesting behavior (Δv) continuously degrades the local vegetation. This physical clearing permanently lowers the repulsive gradient of the corridor. Subsequent agents, driven purely by the static resource utility, are naturally drawn into these freshly cleared topological trenches.

This purely structural feedback loop drives a continuous, month-over-month increase in the Canalization Ratio. The data suggest that even in the absence of explicit chemical or marker-based stigmergy, persistent infrastructural highways can emerge solely through the localized, reciprocal depletion of environmental friction.

#### 3.4.5. Stigmergic Canalization and Phenotypic Asymmetry

To explicitly evaluate the influence of the strategic utility matrices ($U_{x,y}$) on swarm topology, we isolated the model in a high-friction (β=0.8), restricted-payload ($L_{\max}=100$) regime. This configuration ensures that agents are strictly bound by topographic feedback, preventing the stigmergic signal from being masked by unconstrained caloric wandering or low-friction bulldozing.

**Figure 8:**
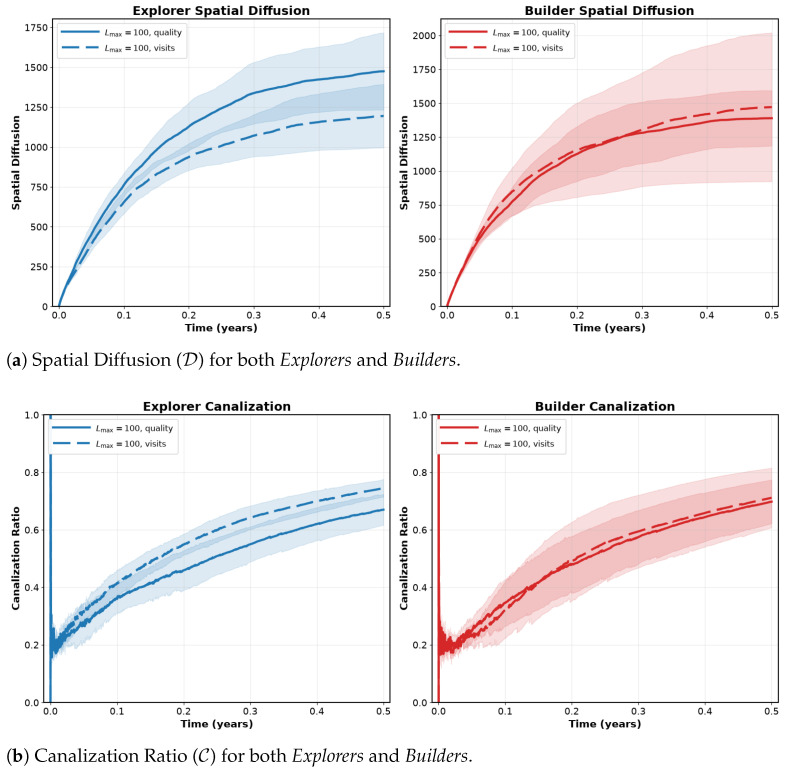
Routing dynamics under parameter constraints ($L_{\max}=100$, β=0.8). Lines represent the Monte Carlo mean (n=5) and shaded bands denote ±1σ variance (shaded areas). (**a**) Spatial Diffusion (D): a restricted payload acts as a physical tether, severely compressing the spatial expansion of both phenotypes. (**b**) Canalization Ratio (C): *Builders* exhibit accelerated path consolidation (C→1), whereas *Explorers* maintain highly diffuse, uncoordinated search trajectories.

As illustrated in Figure 8, introducing the stigmergic ledger (vegetation_visits) triggers a highly asymmetric response between the two phenotypes. For *Explorers*, stigmergic memory acts as a critical spatial anchor. Under resource-driven routing ($U\propto v^{2}$), *Explorers* disperse centrifugally to chase pristine vegetation frontiers, resulting in high diffusion and low path consolidation. Conversely, under stigmergic routing (U∝v·τ), the historical traversal ledger restricts this expansion: agents track established trails, significantly suppressing absolute diffusion while increasing their Canalization Ratio.

Conversely, the *Builder* phenotype is invariant across modes; targeting cleared terrain causes resource utility ($U\propto v^{-2}$) to spike as v→0. This dominant topographic funneling renders explicit stigmergic memory (U∝τ/v) redundant, thereby driving high canalization and indicating that environmental memory primarily alters frontier-seeking phenotypes rather than localized engineers. Finally, the consistent inter-trial variance (±1σ bands) is a signature of the high-friction (β=0.8) regime (Figure 7). High topographic sensitivity causes minor early stochastic deviations in action selection to compound over multi-month integration, thereby maintaining macroscopic variance regardless of the utility matrix.

#### 3.4.6. Trace Persistence and Phenotypic Asymmetry

**Figure 9:**
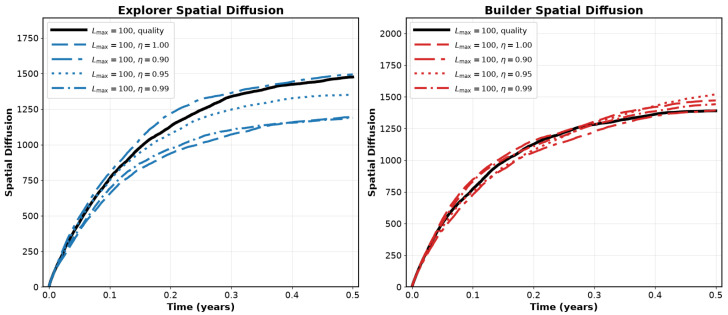
Spatial Diffusion (D) over 6 months ($L_{\max}=100$, β=0.8). Solid black: resource-driven baseline (vegetation_quality); dashed/colored: stigmergic policy (vegetation_visits) across decay rates (η). Variance bands (±1σ) are omitted for clarity but match Figure 7. Results show distinct phenotypic asymmetry: for *Explorers* (**left**), stigmergy suppresses expansion, reverting to baseline at lower η. Conversely, *Builders* (**right**) remain entirely invariant to trace decay, as their topography-altering engineering renders explicit memory redundant.

To isolate the impacts of utility ($U_{x,y}$) and trace persistence (η), we enforce high friction (β=0.8) and low payload ($L_{\max}=100$) to keep unconstrained wandering from masking the signal. We then proceed by sweeping η∈{0.9,0.95,0.99,1.0}. As shown by the Spatial Diffusion (D) in Figure 9, the stigmergic ledger (vegetation_visits) triggers a highly asymmetric phenotypic response.

For *Explorers*, stigmergic anchoring depends on trace longevity. Under resource-driven routing ($U\propto v^{2}$), agents diffuse rapidly to chase pristine frontiers (black baseline). While stigmergic routing (U∝v·τ) with high persistence (η=1.0) successfully restricts expansion, accelerated decay (η=0.90) causes traces to evaporate before reinforcement. In this last case, the utility matrix then degenerates into a basic resource gradient, sending *Explorers* diffusion rapidly back to the baseline.

Conversely, *Builders* remain invariant across the η sweep. Because they target cleared terrain, resource utility ($U\propto v^{-2}$) inherently spikes within harvested patches (v→0). This dominant topographic funneling ensures that even if chemical memory (U∝τ/v) evaporates under low η, the physical trench remains intact, rendering volatile memory redundant for localized engineers.

Finally, though omitted from Figure 9 for legibility, inter-trial variance (±1σ) is stable across all η values, pointing to a distinct signature of the high-friction (β=0.8) regime. High topographic sensitivity causes minor early stochastic deviations in action selection to compound over time, maintaining consistent variance regardless of trace decay.

### 3.5. Cognitive Heuristics and Spatial Stochasticity

While the physical payload capacity ($L_{\max}$) sets the absolute bounds of the swarm’s range, the internal realization of that territory is heavily modulated by the agents’ cognitive heuristics. Biological entities rarely act as perfect mathematical optimizers; their locomotion incorporates spatial stochasticity driven by territorial patrolling, predator evasion, or sub-optimal decision-making. To evaluate this dynamic, we introduced an ϵ-greedy action-selection policy (Section 2.3.2) that dictates the probability of an agent deterministically exploiting a local resource versus stochastically exploring its periphery.

**Figure 10:**
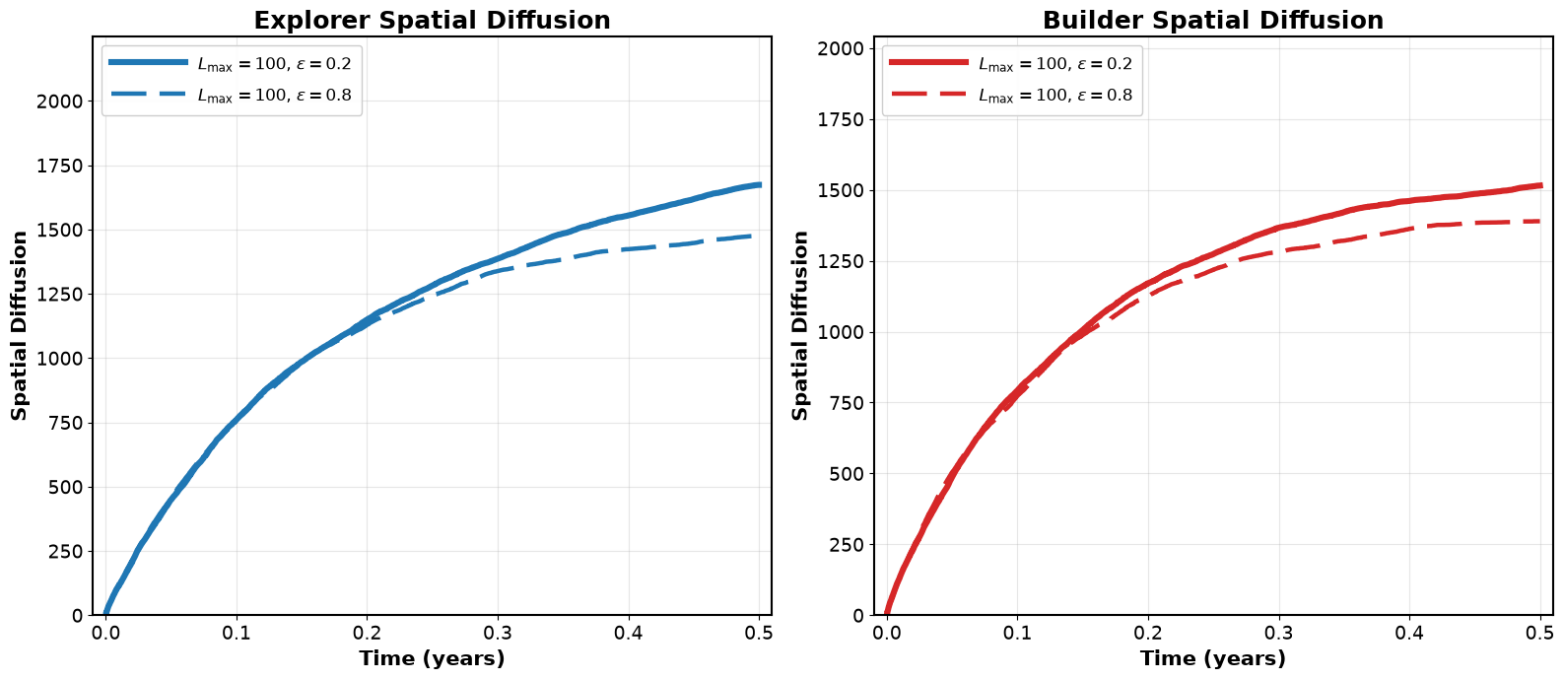
Spatial Diffusion (D) across ϵ-greedy cognitive heuristics ($L_{\max}=100$, β=0.8). Lines and shading denote Monte Carlo mean (n=5). Exploitative focus negatively correlates with spatial diffusion across both phenotypes; however, structural topography strongly dampens the stochastic expansion of *Builders* (red) relative to frontier-seeking *Explorers* (blue).

To evaluate cognitive noise, the Spatial Diffusion (D) was analyzed across an ϵ-greedy sweep (ϵ∈{0.2,0.8}). To isolate this behavioral signal from unconstrained wandering, the environment was locked to the high-friction (β=0.8), constrained-payload ($L_{\max}=100$) regime under the resource-driven routing policy (vegetation_quality). As shown in Figure 10, both phenotypes display a negative correlation between exploitative focus and spatial diffusion. At high exploitation levels (ϵ=0.8), deterministic focus forces agents to systematically exploit local cells before advancing, heavily compressing the spatial diffusion. Conversely, maximizing spatial stochasticity (ϵ=0.2) leads agents to frequently bypass viable local resources, resulting in a diffuse network of fragmented trails. This stochasticity manifests similarly between phenotypes. The increased cognitive noise (ϵ=0.2) allows both the frontier-seeking *Explorers* and the *Builders* to expand their diffusion.

### 3.6. Ecosystem Engineering: Multi-Agent Geomorphological Dynamics

We next evaluate the framework’s capacity to simulate multi-year ecosystem engineering by unfreezing environmental dynamics, activating vegetation mean-reversion, hydrological drift, and stigmergic evaporation (η=0.99). To capture a macro-scale division of labor, the swarm is configured as a heterogeneous colony with an equal split of *Explorers* and *Builders*. To facilitate broad environmental interactions, the tactical obstacle weight is relaxed (β=0.2). The system was simulated over a continuous 4-year integration period.

#### 3.6.1. Ecological Stability and Disturbed Equilibrium

**Figure 11:**
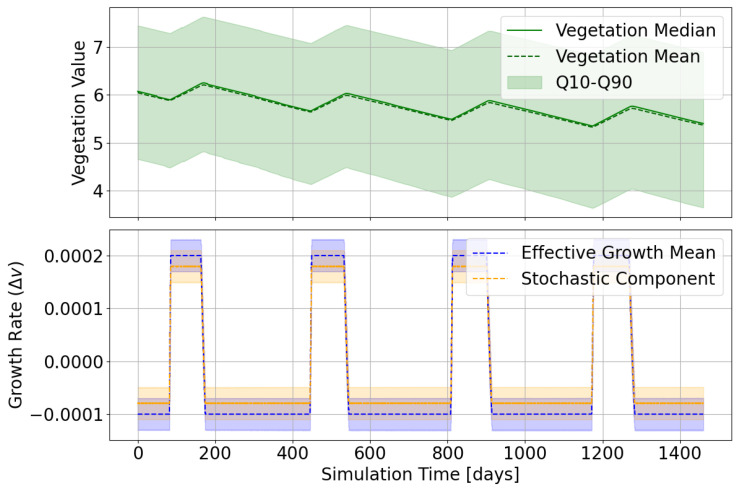
Rumney Ranch vegetation dynamics over a 4-year cycle (n=4). The (**top panel**) shows the stabilization of mean and median vegetation quality. The (**bottom panel**) illustrates the seasonal growth rate, reflecting the periodic activation of the continuous mean-reversion model.

Before analyzing spatial topology, it is necessary to establish the baseline stability of the living environment. The simulation results demonstrate the emergence of a stable, self-regulating feedback loop between agent behavior and environmental dynamics. By maintaining conservative vegetation regrowth parameters, we observed the system’s capacity to sustain long-term ecological equilibrium rather than suffering rapid resource exhaustion. The analysis of the global vegetation layer (Figure 11) confirms this resilience over the 4-year cycle. Despite continuous harvesting activity by the swarm (n=4), the mean and median of the vegetation quality oscillate within a narrow, stable band. The “sawtooth” fluctuations in the growth rate (bottom panel, Figure 11) illustrate the periodic activation of the regrowth mechanism. This pattern confirms that the system operates under a *disturbed equilibrium*; the environment is regulated by a continuous mean-reversion strength (0.0005) that draws the vegetation quality toward a defined annual mean, successfully counteracting the agents’ localized depletion.

#### 3.6.2. Emergent Spatial Patterns and Role Differentiation

**Figure 12:**
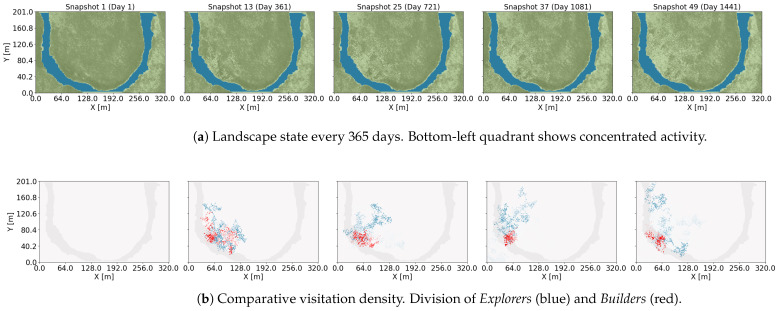
Snapshots of the 4-year spatial evolution for a minimal viable colony (n=4), showing localized environmental depletion and the emergent labor between biological phenotypes.

The dynamic spatial evolution visually supports the phenotypic divergence observed in the targeted parameter sweeps. Over time, snapshots (Figure 12) reveal clear activity distributions driven by lodge proximity and individual utility preferences, showing a stark differentiation in emergent footprints:

- ***Explorers*** **(Blue):** Maintain a highly diffuse distribution. Driven by extended payloads and stochastic boundary-pushing, they serve as a foraging vanguard, tracking shifting frontiers of mature vegetation.
- ***Builders*** **(Red):** Form highly localized, deeply canalized corridors. Constrained by restricted payloads and topographic funneling, they continuously thicken and reinforce the trails initiated by *Explorers*.

#### 3.6.3. Density-Dependent Hydrological Expansion

**Figure 13:**
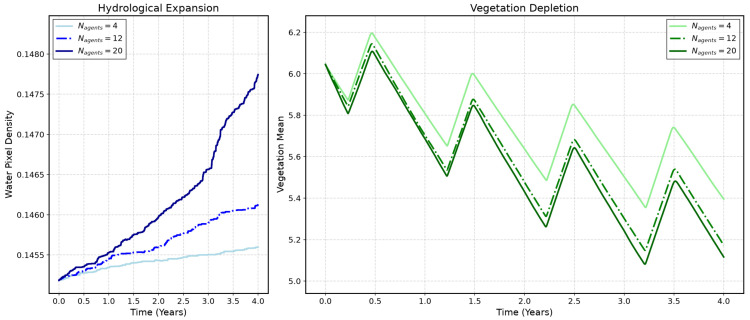
Environmental metrics across colony sizes (Monte Carlo mean, n=5, 4-year cycle). (**Left**): Hydrological footprint suggests a non-linear expansion within the tested colony range. (**Right**): Density-dependent vegetation (*v*) depletion establishes degraded equilibria alongside seasonal regrowth.

**Figure 14:**
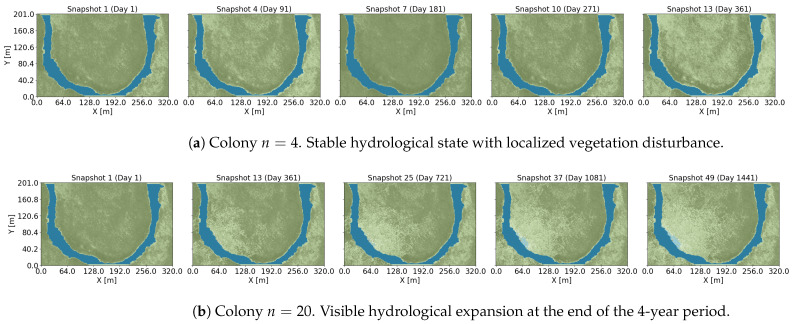
Comparative geomorphological evolution across colony sizes. The extended colony (**a**,**b**) visually demonstrates the transition from localized harvesting to large-scale hydrological engineering, as characterized by the emergence of an auxiliary pond.

To evaluate carrying capacity, we analyzed structural responses across colony sizes (n∈{4,12,20}) with restricted *Builder* payload ($L_{\max}=100$) and a 0.001 structural decay. The tracking of the percentage of water pixels and the vegetation mean (Figure 13) suggests an apparent non-linear transition within the tested colony-size range, with the n=12 case showing a notably stronger response than n=4. Terrestrial vegetation (right panel) degrades proportionally with agent density, establishing lower equilibria with preserved seasonal oscillations. The hydrological impact, instead, scales non-linearly (left panel). The n=4 colony lacks sufficient aggregate *Builder* capacity to overcome environmental resistance, resulting in marginal water expansion. At n=12, the model begins to show a more pronounced geomorphological response: continuous bank clear-cutting activates dynamic hydrology, driving a sustained expansion of the water network within the tested range. Visualized in Figure 14, this macroscopic transition omits explicit dam construction; the auxiliary pond expansion (n≥12) emerges entirely from continuous vegetation removal, stigmergic trampling, and localized bank erosion. Ecologically, this canalization is consistent with the expansion of the Beaver Dam Complex (BDC). Larger swarms carve aquatic transport corridors to safely forage deeper terrestrial zones while minimizing metabolic costs and predation risks, indicating that decentralized, non-linear exploitation can optimize landscapes for long-term colony survival.

## 4. Conclusions

This study introduces a bio-inspired multi-agent framework designed to simulate the localized environmental impacts of the North American beaver (*Castor canadensis*). By coupling a physical engine with decentralized stigmergic navigation and a hydrological proxy, the model demonstrates how complex landscapes emerge from simple behavioral rules. The integration of specialized ecological roles (*Explorers* and *Builders*) successfully generated non-linear, density-dependent landscape modifications. Notably, the framework avoids explicit hard-engineering instructions. Instead, continuous vegetation removal and localized bank trampling naturally drive the expansion of auxiliary canal networks, closely mirroring real Beaver Dam Complexes.

### 4.1. Limitations of the Current Framework

While the emergent topologies align with broad ecological observations, the current framework possesses notable limitations that define it as a foundational computational architecture rather than a predictive model. Acknowledging these constraints is critical:

- **Simplified Hydrology:** To maintain computational efficiency, the environmental hydrology is formulated as a conceptual proxy. The model deliberately abstracts complex fluid dynamics, sediment transport, and dam-induced backwater effects, focusing solely on topological bank erosion.
- **Parameter Sensitivity:** The simulation relies on highly coupled variables governing vegetation growth, trace decay, and agent carrying capacities. These were calibrated to establish a stable baseline proof-of-concept, meaning a comprehensive parameter sensitivity analysis remains for future work.
- **Empirical Validation:** Although internal consistency has been rigorously evaluated via Monte Carlo trials, the simulated spatial footprints have not yet been strictly cross-validated against high-resolution empirical geospatial data.

### 4.2. Future Work: Empirical Extraction and Network Formalization

To facilitate large-scale comparative studies, our next step is transitioning from localized simulations to an automated empirical extraction pipeline. Drawing on recent advancements in the semantic segmentation of large herbivore grazing trails [31], we will investigate using Deep Neural Networks to isolate biological trail networks from high-resolution imagery. This pipeline aims to leverage crowdsourced ecological archives, such as the citizen-science *Beavers from Space* [32] datasets, to train robust extraction models.

Once extracted, these empirical traces and our simulated stigmergic ledgers must be formalized for rigorous comparison. Future iterations will abstract both datasets into discrete Spatial Ecological Networks (SEN). Using the Geographic Network Automata (GNA) framework [33,34], this approach treats the evolving landscape as a dynamic spatial graph where nodes represent key biological states (such as lodges and harvested patches) and edges represent heavily trafficked transit canals. By applying formal graph-theoretic metrics like *k*-degree distribution and betweenness centrality, we can quantitatively evaluate the structural overlap between our simulation and real-world ecosystems. Finally, to advance physical accuracy, future work will couple the multi-agent system directly with grid-based landscape evolution toolkits like Landlab, replacing our conceptual proxy with rigorous hydrological drift and sedimentation dynamics.

## Figures and Tables

**Table 1:** Mapping of computational environment parameters to hydrological dynamic coefficients.

Code Parameter	Symbol	Physical Interpretation
river_growth_interval	[c,d]	Domain of water deepening (e.g., [−3,0])
river_growth_velocity	$\nu_{r}$	Constant rate of channel deepening ($h^{-1}$)
streams_depth	$w_{s}$	Maximum depth for water channels

**Table 2:** Agent Cognitive and Behavioral Parameters.

Parameter	Symbol	Function
maximum_load	$L_{\max}^{\mathrm{init}}$	Initial biomass load.
harvest_interval	H	Vegetation bounds.
epsilon_greedy	$\epsilon_{\mathrm{greedy}}$	Gating threshold.
role	–	*Explorer/Builder*.
decay_values	[·,·]	Decay rates during stall.

**Table 3:** Dynamic Control and Model Parameters.

Parameter	Symbol	Function
mass	*m*	Damping mass.
friction	μ	Viscous damping.
PID Gains	$K_{p},K_{i},K_{d}$	Control coefficients.
beta_repulsive	β	Penalty weight.

**Table 4:** Environmental and Stigmergic Coupling Parameters.

Parameter	Symbol	Function
vegetation_removal	Δv	Harvested mass.
visit_increase	Δτ	Traffic marker.
exploration_map	–	Toggle target mode.
map_repulsive	–	Toggle APF friction.
flow_info (ENV)	$\hat{f}$	Flow vector.
visit_reset (ENV)	η	Ledger decay.

**Table 5:** Summary of Mathematical Models and Biological Equivalents.

Mechanism	Model/Logic	Biological Proxy
Agent Locomotion	APF + PID (Equation (6))	Navigation & drift (λ).
Target Selection	Softmax (Equation (5))	Resource & trail routing.
Role Alternation	Gradient Inversion ($v^{-2}$)	Foraging vs. engineering.
Stigmergic Trailing	Exponential Decay	Pheromone reinforcement.
Vegetation Dynamics	Mean-Reverting SDE	Seasonal regeneration.
Hydrology	Depth Clipping	Bank erosion proxy.

**Figure A1:**
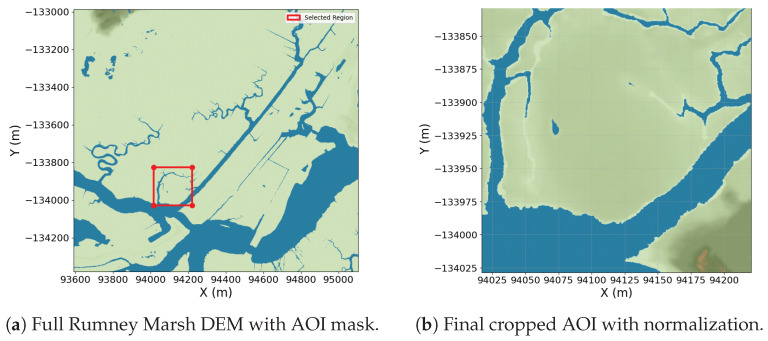
Rumney Marsh processed DEM. (**a**) AOI definition on the projected DEM domain; (**b**) final cropped, normalized grid (1 m res.). The colorbar maps land from green to brown, and water in blue.

## Data Availability

The simulation framework developed in this study is publicly available in the BeaverSim repository at https://github.com/beaverbotassistance/BeaverSim (accessed on 16 July 2026). The raw geospatial datasets (DEMs and RGB imagery) analyzed during this study are publicly accessible through the United States Geological Survey (USGS) EarthExplorer and the National Oceanic and Atmospheric Administration (NOAA) repositories.

## Appendix A. Empirical Data Processing and Calibration

As introduced in Section 2.1, the multi-agent simulation framework is designed to be data-agnostic. Rather than fusing disparate data types, it provides researchers with two independent initialization pathways to generate the computational environment depending on the available field data. This appendix details the computer vision and calibration methodologies required to process both the Digital Elevation Model (DEM) and RGB aerial imagery pipelines into the standardized N×M simulation matrix. Prior to simulation initialization, the raw data are spatially synchronized. This involves reprojecting the imagery using its embedded Coordinate Reference System (CRS) metadata to ensure absolute spatial alignment before mapping them to the multi-agent grid.

### Appendix A.1. DEM-Based Processing and Hydrological Routing

The DEM pipeline ingests georeferenced CSV elevation points and resamples them onto a regular grid via interpolation. To recover the hydrological network in datasets lacking water-class annotations (such as the Acadia site), a percentile-based thresholding step is currently applied. Let *Z* be the elevation field and $P_{q}\left(Z\right)$ its *q*-th percentile. A water mask W(x,y) is generated where:

(A1) $$W\left(x,y\right)=\left\{\begin{array}{cc} 1, & Z\left(x,y\right)\leq P_{q}\left(Z\right)\\ 0, & \mathrm{otherwise} \end{array}\right.\;.$$

The parameter *q* is specific to each dataset and must be calibrated to ensure channel continuity while avoiding over-detection in low-lying areas. While we acknowledge that this rudimentary thresholding lacks the physical rigor of standardized flow-accumulation models, it provides a sufficiently continuous spatial boundary to test the core multi-agent stigmergic behaviors presented in this foundational work. The execution of this spatial interpolation is available via dedicated Jupyter notebooks in the project repository.

This process is illustrated here using the Rumney Marsh and Acadia sites. For Rumney Marsh, water pixels are already labeled in the CSV file; the recovery of the channel network is shown in Figure A1, where the initial Area of Interest (AOI) selection is compared with the final normalized matrix. The sensitivity of the threshold *q* is demonstrated on the Acadia dataset in Figure A2, which shows the progression from conservative to aggressive stream masks. The final matrix maps land to [0,1] and water bodies to [−1,0).

### Appendix A.2. RGB-Based Processing

The RGB pipeline utilizes a multi-temporal aggregation strategy to mitigate the higher noise levels inherent in optical imagery. A baseline is constructed by aggregating multiple images of the same geographic area at different times and under varying lighting conditions. These images are merged into a reference baseline using either pixel-wise median or mean filtering (user-defined) to attenuate transient artifacts such as moving shadows or clouds. To ensure computational repeatability, this iterative multi-temporal aggregation is managed via a batch-processing Jupyter notebook governed by a single .yaml configuration file, allowing researchers to replicate the calibration across large image sets.

To delineate land from water, the framework supports indices like the Excess Green index (ExG), the Normalized Difference Vegetation Index (NDVI) visible approximation, and the Color Water Index (CWI). The user can visualize these indices and their distribution histogram to select the thresholded index that best separates water from land for a given site. Target images are shadow-corrected and histogram-matched to the baseline to ensure index consistency. This methodology is applied here to a specific area of the Rumney Ranch site (referred to as A2), as shown in Figure A3. Specifically, we consider a set of 10 images of the A2 region spanning June to August 2018, using ExG to identify water and generate the final vegetation-quality matrix (see the full image set in the project repository).

A comparison between the shadow-corrected baseline (Figure A3a) and the original target image (Figure A3b) shows the necessity of the multi-temporal approach; the single target acquisition suffers from non-uniform illumination, particularly in the bottom-right corner. Furthermore, the normalized ExG matrices validate the pipeline’s ability to resolve fine biological features. Both in the original target image (Figure A3b) and the final simulation-ready grid (Figure A3d), faint trail tracks are visible in the bottom-left quadrant.

## Appendix B. Vegetation Dynamics Formal Derivation

As introduced conceptually in Section 2.2.1, the vegetation regeneration within the simulation is modeled as a spatially-distributed, seasonally-modulated stochastic process. This appendix details the formal Stochastic Differential Equations (SDEs) and the closed-loop restorative feedback mechanisms that govern these dynamics, ensuring the landscape maintains a stable, yet locally noisy, carrying capacity. At each simulation step, the state $v_{x,y}$ of a cell (x,y)∈E is updated according to:

(A2) $$v_{x,y}\left(t+\Delta t\right)=\mathrm{clip}\left(v_{x,y}\left(t\right)+\Delta v_{x,y}\left(t\right),\;v_{\min},\;v_{\max}\right),$$

where the absolute vegetation state is strictly bounded to the interval $\left[v_{\min},v_{\max}\right]$ to prevent collapse or infinite overgrowth. The increment $\Delta v_{x,y}\left(t\right)$ is sampled for each cell within the valid growth interval [a,b] via the indicator function $I_{\left[a,b\right]}$:

(A3) $$\Delta v_{x,y}\left(t\right)∼N\left(\mu_{\mathrm{eff}}\left(t\right),\sigma_{v}^{2}\left(x,y\right)\right)\cdot I_{\left[a,b\right]}\left(v_{x,y}\left(t\right)\right).$$

To capture the natural variety of the landscape, the plant growth variance ($\sigma_{v}^{2}\left(x,y\right)$) varies with location. We first define a baseline variability $\sigma_{\mathrm{base}}=\sigma_{0}\left|\gamma \right|$, where $\sigma_{0}$ is a configurable noise parameter and γ is the baseline growth rate. This baseline is then amplified locally: growth becomes more variable in rough patches where a cell’s vegetation contrasts heavily with its surroundings. Computationally, we balance out these random changes across the map at each time step (zero-centering) so that the total amount of vegetation does not accidentally drift over time. This ensures that the macroscopic health of the landscape is controlled solely by the effective mean growth rate, $\mu_{\mathrm{eff}}\left(t\right)$:

(A4) $$\mu_{\mathrm{eff}}\left(t\right)=\mathrm{clip}\left(\mu_{\mathrm{base}}\left(t\right)+k\left(v_{\mathrm{target}}\left(t\right)-\bar{v}\left(t\right)\right),\;\mu_{\min},\;\mu_{\max}\right).$$

Here, $\bar{v}\left(t\right)$ is the spatial average of the vegetation across the growth mask. The parameter *k* acts as the proportional gain, creating a feedback that pulls the landscape’s average toward a moving seasonal target, $v_{\mathrm{target}}\left(t\right)$. The growth bounds are defined relative to the base rate γ as $\mu_{\min}=-\gamma$ and $\mu_{\max}=2\gamma$, allowing the restorative feedback to temporarily exceed standard vernal growth when recovering from severe depletion. The target state $v_{\mathrm{target}}\left(t\right)$ is a continuous linear interpolation between the landscape’s winter minimum and its summer peak. In this framework, $v_{\mathrm{winter}}$ and $v_{\mathrm{summer}}$ are defined as ±20% of the environment’s initial spatial mean, anchoring the feedback loop to the site’s original carrying capacity. Both $\mu_{\mathrm{base}}\left(t\right)$ and $v_{\mathrm{target}}\left(t\right)$ are modulated by a normalized seasonal drive S(t), ensuring that the restorative feedback tracks the vegetation cycle:

(A5) $$\begin{array}{cc} \mu_{\mathrm{base}}\left(t\right) & =\gamma \left(2S(t)-1\right), \end{array}$$

(A6) $$\begin{array}{cc} v_{\mathrm{target}}\left(t\right) & =v_{\mathrm{winter}}+\left(v_{\mathrm{summer}}-v_{\mathrm{winter}}\right)S\left(t\right). \end{array}$$

To capture the asymmetry of rapid vernal growth and gradual autumnal decay, S(t) is defined using the probability density function (PDF) of a skew-normal distribution. Let τ∈[0,364] represent the day of the year. The seasonal drive is normalized to its peak as:

(A7) $$S\left(t\right)=\frac{f_{\mathrm{SN}}\left(\tau ;\;\xi ,\omega ,\alpha \right)}{f_{\mathrm{SN}}\left(\xi ;\;\xi ,\omega ,\alpha \right)},$$

where $f_{\mathrm{SN}}$ is the skew-normal PDF:

(A8) $$f_{\mathrm{SN}}\left(\tau ;\;\xi ,\omega ,\alpha \right)=\frac{2}{\omega }\phi \left(\frac{\tau -\xi }{\omega }\right)\Phi \left(\alpha \frac{\tau -\xi }{\omega }\right).$$

In this formulation, ϕ and Φ are the probability density and cumulative distribution functions of the standard normal distribution, respectively. The parameters define the site’s biological rhythm: ξ is the peak growth day, ω controls the seasonal window width, and α>0 defines the right-skewness necessary to model slow autumnal decay. This model ensures that while individual cells exhibit realistic, contrast-driven local stochasticity, the macroscopic landscape preserves a stable, seasonally oscillating topological memory. To facilitate reproducibility and bridge these formal mathematical derivations with the open-source software implementation, Table A1 summarizes the direct mapping between the computational environment parameters (configurable in the simulation backend) and the state-space dynamic coefficients defined above.

**Table A1:** Mapping of computational environment parameters to state-space dynamic coefficients.

Code Parameter	Symbol	Physical Interpretation
vegetation_quality_range	$\left[V_{\min},V_{\max}\right]$	Environmental state’s bounds
grass_growth_interval	[a,b]	State domain for passive regeneration
grass_growth_rate	γ	Baseline maximum growth rate ($h^{-1}$)
grass_growth_sigma	$\sigma_{0}$	Scaling factor for micro-climatic effect
mean_reversion_strength	*k*	Gain of the restorative feedback
